# Impact of small municipal solid waste landfill on groundwater quality

**DOI:** 10.1007/s10661-019-7279-5

**Published:** 2019-02-19

**Authors:** Grzegorz Przydatek, Włodzimierz Kanownik

**Affiliations:** 1 Engineering Institute, State University of Applied Sciences in Nowy Sącz, Zamenhofa 1a St., 33-300 Nowy Sącz, Poland; 2 Faculty of Environmental Engineering and Land Surveying, University of Agriculture in Krakow, Ave. Mickiewicza 24-28, 30-059 Kraków, Poland

**Keywords:** Landfill site, Waste, Groundwater, Leachate, Pollution

## Abstract

The aim of this paper is to analyse changes in the physicochemical elements in groundwater in the vicinity of a small municipal solid waste landfill site located within the territory of the European Union on the basis of 7-year hydrochemical research. Samples of groundwater and leachate near the examined landfill were collected four times a year during two periods, between 2008 and 2012 during the use of the landfill and between 2013 and 2014 at the stage of its closure. The research results were analysed on the basis of general physicochemical properties: pH; total organic carbon (TOC); electrical conductivity (EC); inorganic elements: Cr, Zn, Cd, Cu, Pb, Hg; and one organic element—polycyclic aromatic hydrocarbon (PAH). The analysis was carried out in accordance with the EU and national legislation requirements regarding landfill monitoring. The assessment of the groundwater and analysis indicators of the leachate pollution allowed interpretation of the impact of the municipal solid waste landfill on the state of the water environment in the immediate vicinity*.* The results show that the increased values of Cd, EC, and TOC turned out to be the determinants of the negative impact of leachate on the groundwater quality below the landfill. The integrated water threat model determined the potential negative impact of a landfill site. The extent depended on local environmental conditions and the self-cleaning process. Deterioration of the chemical status in the quality of the groundwater within the landfill area was a consequence of the lack of efficiency of the existing drainage system, which may result from the 19-year period of its use. The applied correlation relationship between physicochemical elements between leachate and groundwater with a time shift due to the extended time of migration of contaminants or mass transfer in waterlogged ground can be an important tool to identify the threat of groundwater pollution in the area of landfill sites.

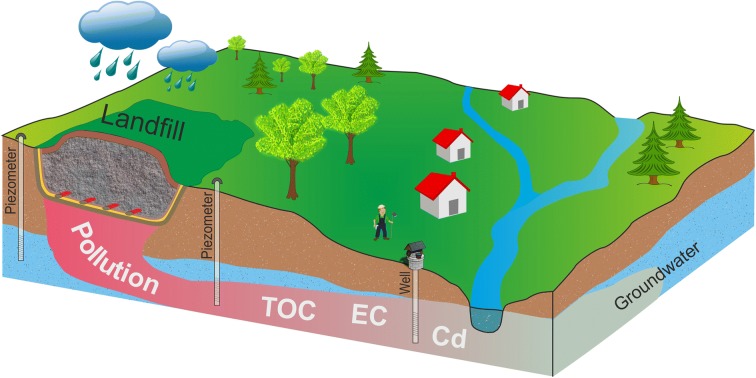

## Introduction

Waste storage is still the most common waste-management method in the world (Laner et al. 2012). Because of the dominance of this form of waste management in Poland (Koda et al. 2017) due to membership in the European Union*,* particular attention is paid to the improvement of municipal waste management. In connection with the guidelines contained in Directive of the Council No. 1999/31/EC of 26 April 1999, enforcement of the environmental regulations concerning the monitoring of pollution in the area of landfill sites is required, including in particular the water environment, including the leachate. Such commitment in EU countries also involves monitoring of the landfill sites in both the operational and post-operational phases (Magrinho et al. 2006). Leachate constitutes a complex matrix of various chemical substances, including dissolved organic matter, inorganic salts, organic trace impurities, and heavy metals, each of which appears in different concentrations due to the physical, chemical, and microbiological processes taking place in the deposited waste (Aziz et al. 2010). Many factors influence the leachate composition, among them the type of deposited waste, the method of exploiting the landfill, and the availability of oxygen, as well as the hydrogeological conditions and the age of the landfill (Chofqi et al. 2004; Regadío et al. 2012). Inspection of a municipal waste landfill site is advisable due to the potential threat that leachate poses to the surrounding environment, including in particular the groundwater (Ağdağ and Sponza 2005; Xie et al. 2015) as a consequence of its infiltration by the deposited waste (Brennan et al. 2016). Many researchers have investigated the impact of landfill sites on the quality of groundwater (Dhere et al. 2008) and have found the groundwater to be polluted by leachate. The risk of pollution of the groundwater by the leachate from the landfill sites is considered to be the most significant risk for the natural environment and human health related to the tipping of waste (Kjeldsen and Christophersen 2001; Deshmukh and Aher 2016). This threat is harmful to both surface and groundwater sources and results from the toxicity of the leachate (Koshy et al. 2007), and its subsequent migration, which is a serious problem of environmental pollution (Gavrilescu 2004). Minimization of groundwater contamination below the landfill should be ensured by the leachate collection system (VanGulck and Rowe 2004) and reinforced by suitable operation of the landfill site. An appropriate operation of a landfill site, maintaining the leachate at the lowest level possible, is the key element of the protection of the water environment neighbouring landfill sites. This is important in the context of treatment of groundwater as a major source of water supply in both urban and rural areas (Singh et al. 2016).

The aim of this study was to identify the changes in the physical and chemical components of groundwater on the basis of the 2008–2014 studies in the area of a small landfill site situated in G in the territory of the European Union, taking into account the phases of operation and closing of the landfill.

## Materials and methods

**Fig. 1:**
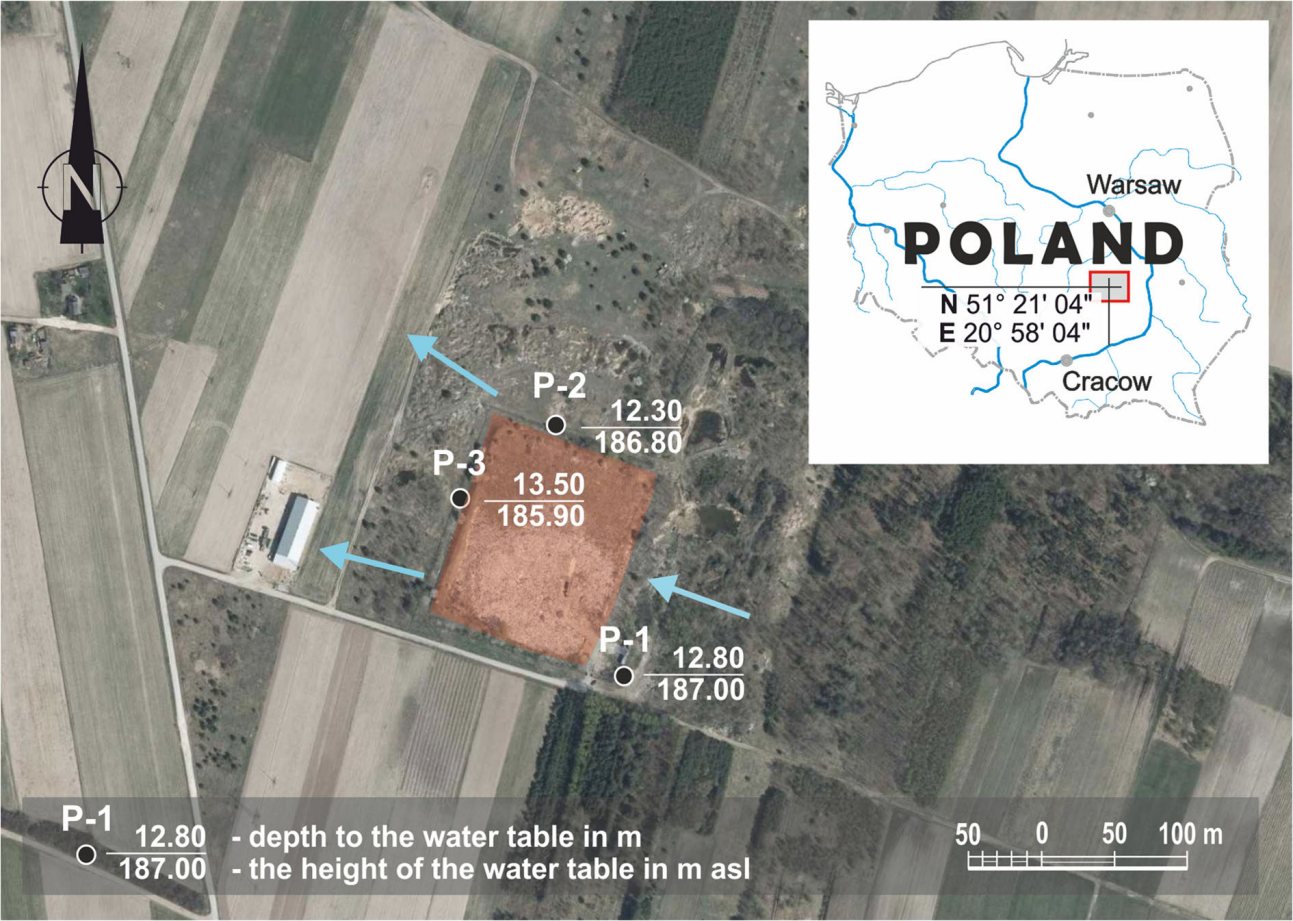
The location of piezometers

Groundwater and leachate were taken from the piezometers situated around the landfill site in G (in central Poland) and from the collective wells at the landfill site quarterly during 2008–2014. Groundwater samples for laboratory testing were taken after prior pumping of water out of the piezometers: P-1, situated in front of (above) the landfill site, served the purpose of monitoring the quality of the water flowing into the area of the landfill site; water from this piezometer formed the reference point of the water quality (the so-called background) and two piezometers, P-2 and P-3, situated in the vicinity of the landfill site on the side of the outflow of the groundwater, serving the purpose of identification of the concentration of pollutants below the landfill site (Fig. 1). Water from piezometer P-2 had been taken since 2008 until July 2012 when the landfill site was closed. Leachate from the peripheral drainage system situated along the slope at the bottom of the landfill site trough was taken from the collective well situated in its bottom part.

An analysis of the composition of the leachate from the landfill site at G together with that of the groundwater covered three general physiochemical characteristics: the pH value, total organic carbon (TOC), electrical conductivity (EC), and six inorganic elements: hexavalent chromium (Cr), zinc (Zn), cadmium (Cd), copper (Cu), lead (Pb), mercury (Hg) and one organic element: polycyclic aromatic hydrocarbon (PAH).

The reaction and electrical conductivity was identified immediately on site after collection of the samples of groundwater and leachate in the area of the landfill site. To this end, a portable multifunction metre with glass electrodes was used after prior calibration of its electrodes. Other analyses of chemical compounds were performed at an accredited laboratory after prompt delivery thereto (within 48 h of the samples being taken) (APHA 2007). Heavy metals, such as Cu, Cd, Cr^+6^, Pb, Zn, and Hg, were determined by the AAS method (atomic absorption spectroscopy). The results of the tests of the leachate from the landfill site were compared with the values listed in the Regulation of the Minister of Environment of 18 November 2014 concerning the Conditions that should be Met upon Introduction of Wastewaters to Water or to the Ground (Regulation ME 2014). The quality of the groundwater in the analysed piezometers was determined in accordance with the Regulation of the Minister of Environment of 21 December 2015 concerning the Criteria and Methods of Assessment of the Groundwater Bodies (Regulation ME 2015).

Static parameters were determined for the contamination factors in the leachate: minimum and maximum values, arithmetic mean, and standard deviation. For the purpose of calculation of the static parameters, the measurement result at the level of one half of the specific limit of quantification was adopted if the values of the water indices in a specific sample fell below the limit of quantification determined by the multiple of the limit of detection, i.e. the output signal or the concentration limit above which a sample, with a stated level of confidence, may be pronounced as different from the blank sample. The aggregate results of the physiochemical elements of the condition of the groundwater from the piezometers were used to perform a statistical analysis and to draw conclusions aimed at the assessment of the influence of the landfill site on the quality of the groundwater in the immediate vicinity. A non-parametric Kruskal-Wallis test and multiple (bilateral) comparison test of the mean ranges, which do not require a normal distribution or homogenous variances, were applied for assessment of the importance of the difference in the concentration of the tested groundwater parameters above and below the municipal landfill site. Non-parametric tests were applied because the distribution of the most of the analysed physiochemical characteristics was abnormal in accordance with the results of the Shapiro-Wilk test, and the variances were not equal, as determined using Fisher-Snedecor’s test. For specific elements which significantly differed between the points, extreme values, the median, and the interquartile range were presented in box plots.

In order to determine the impact of the municipal landfill site on the physiochemical condition of the groundwater, correlations between the physiochemical elements of the leachate and the water taken from the piezometers situated below the landfill site were determined. Each correlation was calculated using Spearman’s range method, which yields a correlation index that is a non-parametric equivalent of the respective Pearson’s index. The range correlation can show any monotonic dependence (including in particular a non-linear one). As in the case of a parametric correlation, the Spearman range correlation index measures the force of the co-dependencies between the variables; however, no quantitative scale of normal distribution is required in this case. The correlations of the physiochemical characteristics between the leachate and water in the piezometers were calculated in three variations. In the first variation, results from specific test dates were compared. In two subsequent variations, the results for the leachate were compared with the water in the piezometers with a time shift of one or two calendar quarters due to the extended time of migration of the pollution or transport of the mass in a hydrated land site, since leachate constitutes a significant source of contamination of groundwater for the foreseeable future (Mor et al. 2006; Ritzkowski and Stegmann 2007).

The statistical analyses were performed using the Statistica 12 software developed by StatSoft.

## Characteristics of the examined object

The landfill site is in G (south of the Mazowieckie Voivodship in Poland) (Fig. 1) as is situated within the macroregion of the Kielce Highland (*Wyżyna Kielecka*) in the mezoregion of the Iłżeckie plateau (*Podgórze Iłżeckie*). It is near an asphalt-paved road approximately 450 m south of a railway line and 3 km north of residential settlements. The average amount of rainfall in the analysed area is 468 mm, which level was recorded at the weather station in Łaziska located 4 km from the landfill site.

There are no watercourses in the immediate vicinity of the site. The landfill site comprises a single accommodation which, due to its situation at a step in the ground level, has a sub-/supralevel form. The shape of the landfill site approximates a rectangle with dimensions 130 × 165 m, with a surface area of approx. 2.04 ha, of which the accommodation takes up 1.30 ha. The area of the landfill site provides for an opportunity to deposit waste of maximum volume 51,350 m^3^. The average thickness of the waste deposited during the last period of operation of the landfill site is 2.5 m. The landfill site was operated in 1993–2012 using the horizontal method consisting in layering the waste in layers 1-m thick, which layers were further divided into working stripes. Waste was unloaded by daily working parties and subsequently transferred and mechanically densified using heavy-duty equipment which allowed for the creation of thin intermediate layers from 30 to 50-cm thick. The landfill site has its bottom and inside escarpments sealed using a layer of clay 0.5-m thick. The substratum of the landfill site comprises surface deposits belonging to quaternary formations developed in the form of glacial tills which provide good insulation for the deeper layers of the soil. The borings performed up to 6-m deep showed that nearly all of these formations consist of hardly permeable deposits. The aquifer in the area of the analysed facility is approx. 12-m deep and is present in the marls and Upper Cretaceous limestones. The groundwater in the area of the landfill site flows in a south-eastern to a north-western direction.

The landfill site in question is equipped with a peripheral drainage system made of ceramic filters with a diameter of 15 cm. It is situated along the slope at the bottom of the trough. Over the mineral sealing, a drainage system was placed with a view to collecting the precipitation waters permeating the deposited waste. The leachate were discharged via the drainage system to the collective wells. When the landfill site was in operation, a portion of the leachate was used to sprinkle the waste while the excess was taken to a waste water treatment plant.

## Results

### Quantitative analysis of deposited waste

In 2008–2012, the amount of municipal waste deposited in the rural landfill site at G ranged from 2974 Mg to 1,082 Mg year^−1^, with an average level of 1443 Mg. During the last 5 years of operation of the site, the amount of deposited waste was at the level of 7215 Mg, with a conspicuous drop in the amount (Table 1). Generally, during the entire cycle of operation of the landfill site, the approximate amount of deposited waste was 23,924 Mg, which means 5 Mg day^−1^ of waste over 19 years. Waste was disposed of in the landfill site from several rural communes. No recovery of waste with a view to limiting the amount of deposited waste was carried out at the site.

**Table 1:** The amount of municipal waste deposited in the landfill site with statistical parameters

2008	2009	2010	2011	2012	Min.	Max.	Average
Amount of waste (Mg)							
2974	1654	711	794	1082	711	2974	1443

### Qualitative analysis of leachate and groundwater in the municipal landfill area

During the tests which had been conducted for a period of 7 years, the concentration of substances in the leachate that are particularly harmful for the water environment was found to be a few times lower than the highest admissible value (Regulation ME 2014). The concentration of mercury did not exceed 3 μg L^−1^, the maximum concentration of cadmium amounted to 37 μg L^−1^ (Table 2). Among the remaining indices of pollution, the pH value of the leachate was higher than the highest admissible value (pH 9) three times only. The maximum value of pH 9.4 was ascertained in the second quarter of 2008. At the beginning of tests in 2008 and 2009, the pH level of the leachate was elevated; however, in the following years, it did not exceed a pH of 8. Leachate from the landfill site did not meet the requirements allowing for the discharge of waste water to waters or to soil mainly due to the high concentration of total organic carbon. The maximum concentration of total organic carbon amounted to 555 mg C L^−1^, and its average value was 198 mg C L^−1^ during the 7-year period; this was higher by 163 mg than the highest admissible value. Among the tested concentrations of heavy metals, only the concentration of zinc (1.55 mg Zn L^−1^) was close to the limit value. The concentrations of the other metals were at a very low level. The maximum concentration of hexavalent chromium was below 0.06 mg L^−1^, and the concentrations of copper and lead were below 0.2 mg L^−1^.

**Table 2:** Statistical parameters describing values of pollution indicators in the leachate from municipal solid waste landfill and admissible values

Pollution indicators	Min.–Max.	Average	Standard deviation	The highest admissible values in accordance with Regulation ME (2014)
Substance especially harmful to the aquatic environment				
Mercury (μg L^−1^)	< 0.5–< 3	0.65	0.59	30
Cadmium (μg L^−1^)	< 2.5–37	6.2	10.1	200
Other indicators				
pH	6.60–9.40	7.39	–	6.5–9.0
TOC (mg L^−1^)	21–555	198	122	30
Zinc (mg L^−1^)	< 0.002–1.55	0.21	0.35	2
Hexavalent chromium (mg L^−1^)	< 0.008–0.056	0.012	0.011	0.5
Copper (mg L^−1^)	< 0.005–0.16	0.031	0.039	0.5
Lead (mg L^−1^)	< 0.005–0.16	0.033	0.044	0.5
EC (μS cm^−1^)	2350–11,680	6021	2524	–
PAH (μg L^−1^)	< 0.017–1.41	0.253	0.411	–

On the basis of the analysis of the quality of the groundwater in the piezometers situated in the area of the landfill site in G, most of the tested physiochemical elements met the standards of water of very good quality, i.e. first class (Table 3). Over the 7-year period, all of the average values of the physiochemical components determined in the groundwater taken from piezometer P-1 (above the landfill site) were lower than the borderline value for water of first class and also met the norms for the hydrochemical background. Below the landfill site, groundwater in piezometer P-3 was classified as water of good quality, i.e. of the second class, due to a borderline value for cadmium (the average value of the concentration of cadmium was 1.05 μg L^−1^). However, in piezometer P-2, two physiochemical characteristics, i.e. electrical conductivity and total organic carbon, contributed to a deteriorated quality of the water. The conductivity is the index of non-dissolved inorganic ions (Kale et al. 2010), and total organic carbon is the index of dissolved organic matter in the groundwater (Tałałaj and Biedka 2016). In comparison with piezometer P-1, a more than twofold increase in average electrical conductivity was observed (1335 μS cm^−1^) which classifies the water as being second class, as well as a more than threefold increase in the average concentration of total organic carbon, up to a value of 10.6 mg C L^−1^. Concentration of total organic carbon classifies the water into the non-satisfactory quality class (fourth class), where the value of the physiochemical characteristics was increased as a result of natural processes occurring in the groundwater and as a result of evident impact of the anthropogenic factor (Wiśnios et al. 2015). In water taken from piezometer P-2, in comparison with that of piezometer P-1, an over fivefold increase of concentration of cadmium, over fourfold increase in the concentration of lead, and nearly fourfold increase in the concentration of mercury were observed. However, the values were at low levels and did not exceed the borderline values for the first class of quality of the ground waters.

**Table 3:** Scope na average values of physicochemical elements and groundwater quality class

Physicochemical element	Piezometer						Limit value in classes (Regulation ME 2015)				
	P-1	P-2	P-3	P-1	P-2	P-3	Hydrochemical background	I	II	III	IV
	Min.–Max.			Average							
General elements											
pH	7.01–8.30	6.40–7.85	7.10–8.10	7.76	7.39	7.68	6.5–8.5	6.5–9.5			< 6.5 or > 9.5
TOC (mg L^−1^)	1–6.3	< 0.1–20.2	< 0.3–13.2	3.4	10.6	2.6	1–10	5	10	10	20
EC (μS cm^−1^)	231–924	207–2796	204–618	572	1335	441	200–700	700	2500	2500	3000
Inorganic elements											
Hexavalent chromium (mg L^−1^)	< 0.1	< 0.008 –< 0.01	< 0.008–< 0.01	0.0050	0.0046	0.0048	0.00001–0.01	0.01	0.05	0.05	0.1
Zinc (mg L^−1^)	< 0.05	< 0.002–0.088	< 0.002–0.039	0.025	0.030	0.025	0.005–0.05	0.05	0.5	1	2
Cadmium (μg L^−1^)	< 0.3	< 0.3–< 3	< 0.3–10.5	0.15	0.75	1.05	0.1–0.5	1	3	5	10
Copper (mg L^−1^)	< 0.002–0.110	< 0.002–0.018	< 0.002–0.013	0.0076	0.0070	0.0026	0.001–0.02	0.01	0.05	0.2	0.5
Lead (mg L^−1^)	< 0.004–0.004	< 0.004–0.030	< 0.004–0.013	0.0021	0.0091	0.0034	0.001–0.01	0.01	0.025	0.1	0.1
Mercury (μg L^−1^)	< 0.05	< 0.05–< 0.3	< 0.05–< 0.3	0.025	0.081	0.061	0.05–1	1	1	1	5
Organic element											
PAH (μg L^−1^)	< 0.017–< 0.06	< 0.017–< 0.06	< 0.017–< 0.06	0.017	0.022	0.020	0.001–0.1	0.1	0.2	0.3	0.5

### Statistical comparative analysis

In water from piezometer P-3, lower values of the general elements (pH, EC, TOC) were observed as well as lower concentrations of hexavalent chrome and copper in comparison with piezometer P-1. However, a sevenfold increase in the concentration of cadmium was observed, which resulted in deterioration in the quality of water from class I to class II, along with a more than factor 2 increase in the concentration of mercury.

**Fig. 2:**
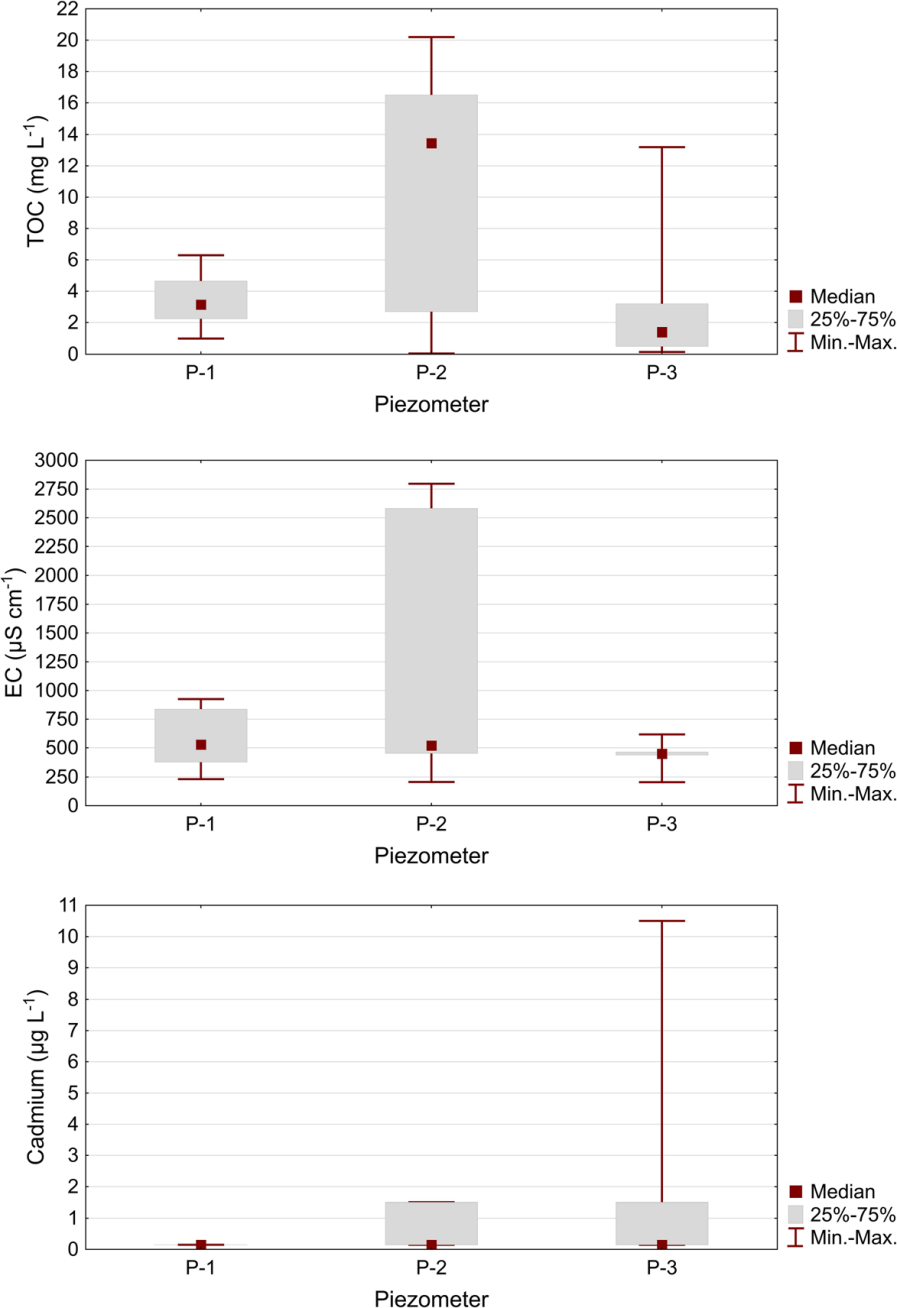
Extreme conditions

Statistical comparative analysis of the physiochemical components in the groundwater, which was carried out using the Kruskal-Wallis non-parameter test, showed that six of the ten tested indices, i.e.: pH, TOC, EC, Cd, Cu, and Pb, differed in the statistical terms among the piezometers (Table 4). The differences occurred between the piezometer situated above the landfill site and the piezometers below the landfill site. Water in P-2 had a pH level significantly lower in statistical terms than that in piezometer P-1, as well as a higher concentration of total organic carbon and higher electrical conductivity than in piezometer P-3 (Fig. 2). Among the inorganic elements in the water, in piezometer P-2, the concentrations of copper and lead were significantly higher in statistical terms than in piezometers P-1 and P-3. Differences in the concentration of cadmium that were significant in statistical terms occurred in piezometers P-1 and P-3, even though the values of the medians were identical; however, the maximum values in individual piezometers differed considerably (Fig. 2).

**Table 4:** Comparison of physicochemical elements values between piezometers using non-parametric Kruskal-Wallis test

Physicochemical element	Piezometer			Results of Kruskal-Wallis test	
	P-1	P-2	P-3	Test value	Probability test (*p*)
	Median				
General elements					
pH	*7.90* ^2 a^	*7.53* ^1^	7.70	*9.60*	*0.008* ^b^
TOC (mg L^−1^)	3.2	*13.5* ^3^	*1.4* ^2^	*12.4*	*0.002*
EC (μS cm^−1^)	*530* ^2^	*722* ^1; 3^	*449* ^2^	*6.77*	*0.03*
Inorganic elements					
Hexavalent chromium (mg L^−1^)	0.005	0.005	0.005	3.51	0.22
Zinc (mg L^−1^)	0.025	0.025	0.025	1.23	0.54
Cadmium (μg L^−1^)	*0.15* ^3^	0.20	*0.15* ^1^	*10.6*	*0.005*
Copper (mg L^−1^)	*0.001* ^2^	*0.005* ^1; 3^	*0.001* ^2^	*25.6*	*< 0.001*
Lead (mg L^−1^)	*0.002* ^2^	*0.005* ^1; 3^	*0.002* ^2^	*16.4*	*< 0.001*
Mercury (μg L^−1^)	0.025	0.025	0.025	2.63	0.31
Organic element					
PAH (μg L^−1^)	0.018	0.025	0.018	5.46	0.07

^a^The digits in the upper index mean a piezometer in which the parameter values are significantly different for multiple (two-sided) comparisons of the average ranks

^b^Statistical values in italic mean statistically significant differences at *p* < 0.05

**Fig. 3:**
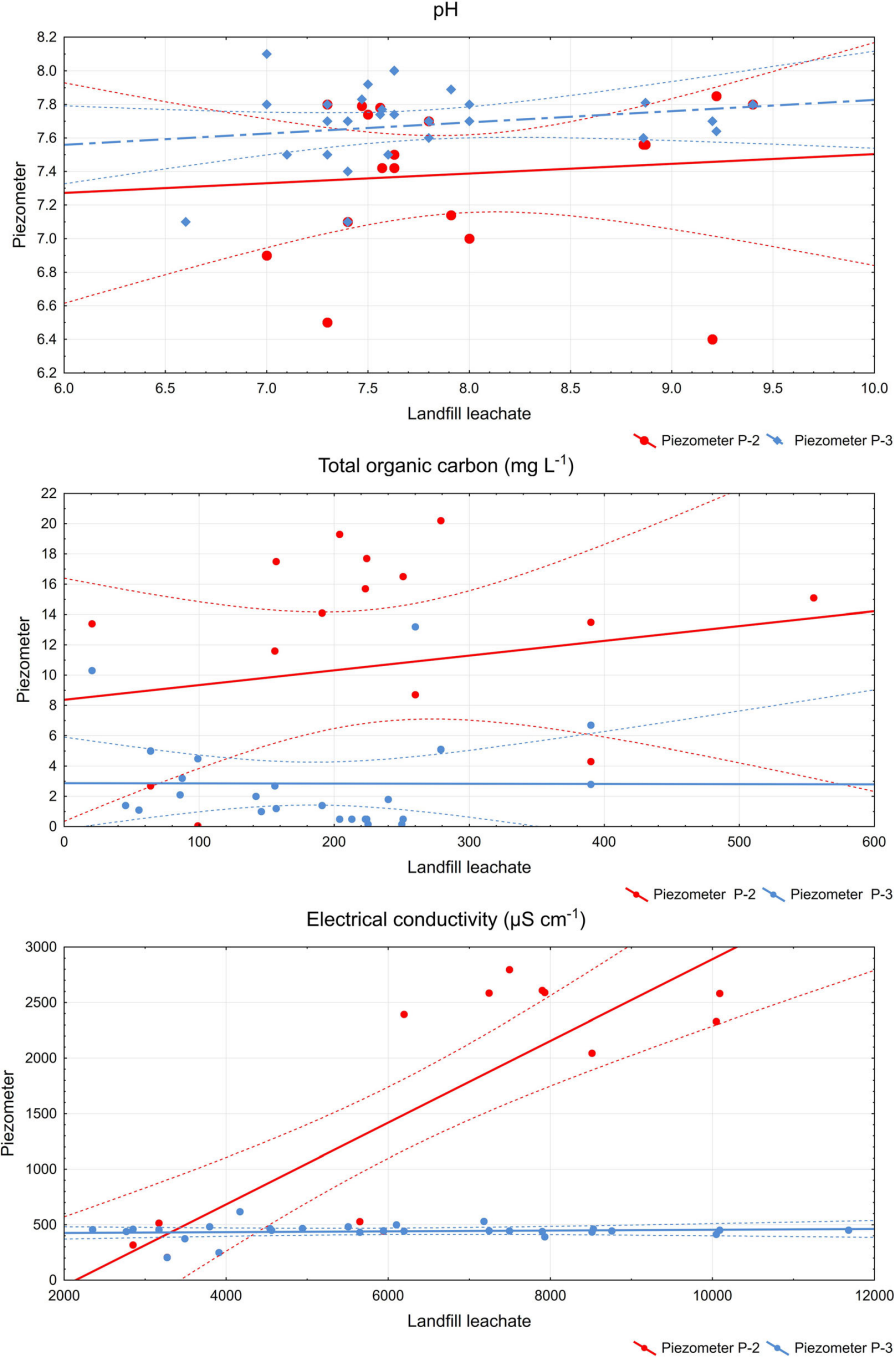
Correlation dependence

In order to assess the impact of the landfill site on the physiochemical condition of the groundwater, the correlation dependence between the water in the piezometers below the landfill site and the leachate from the landfill side was analysed. A statistically important dependence (positive correlation) between the water in piezometer P-2 and the leachate was observed as far as the electrical conductivity (Fig. 3) and concentration of mercury. On the other hand, the water in piezometer P-3 had a statistically important correlation with the leachate as far as the concentrations of cadmium, copper, mercury and polycyclic aromatic hydrocarbons are concerned (Table 5). It follows from the above analysis, that the strongest dependence between the water in the piezometer and the leachate occurs upon comparison of the value of the physiochemical characteristics measured on the same date of intake of the samples. When we compare the results of the tests for the water in the piezometers based on a shift in time by 3 or 6 months (due to the time of migration of contamination in the soil) with the leachate from the landfill site, a drop in the correlation dependence was observed (Spearman’s correlation factor becomes smaller).

**Table 5:** Correlation dependence of physicochemical elements between water in piezometers P-2 and P-3 and leachate from landfill

Physicochemical elements	Piezometer	R Spearman			*t*(*n*-2)			Test probability (*p*)		
		a^a^	b	c	a	b	c	a	b	c
General elements										
pH	P-2	0.190	0.191	0.068	0.776	0.754	0.254	0.45	0.46	0.80
	P-3	0.203	0.180	0.186	1.06	0.913	0.923	0.30	0.37	0.36
TOC (mg L^−1^)	P-2	0.178	0.088	0.399	0.722	0.343	1.63	0.48	0.74	0.13
	P-3	−0.112	0.328	0.095	−0,530	1.59	0.425	0.60	0.13	0.68
EC (μS cm^−1^)	P-2	*0.654*	0.309	0.247	*3.35*	1.26	0.953	*0.004* ^b^	0.23	0.36
	P-3	−0.097	−0.353	−0.119	−0.498	−1.89	−0.587	0.62	0.07	0.56
Inorganic elements										
Hexavalent chromium (mg L^−1^)	P-2	0.099	0.306	0.169	0.399	1.25	0.641	0.69	0.23	0.53
	P-3	0.005	0.58	0.031	0.027	0.798	0.154	0.98	0.43	0.88
Zinc (mg L^−1^)	P-2	−0.071	0.050	0.125	−0.283	0.196	0.471	0.78	0.84	0.64
	P-3	−0.038	−0.151	0.045	−0.193	−0.762	0.219	0.85	0.45	0.83
Cadmium (μg L^−1^)	P-2	−0.182	−0.271	−0.360	−0.738	−1.10	−1.44	0.47	0.29	0.17
	P-3	*0*.*722*	*0*.*584*	*0*.*447*	*5.31*	*3.61*	*2.45*	*< 0.001*	*0.001*	*0.02*
Copper (mg L^−1^)	P-2	−0.160	−0.194	0.139	−0.647	−0.768	0.526	0.53	0.454	0.61
	P-3	*0*.*405*	*0*.*403*	0.353	*2.26*	*2.23*	1.85	*0.03*	*0.04*	0.08
Lead (mg L^−1^)	P-2	−0.032	0.046	0.073	−0.128	0.180	0.275	0.90	0.86	0.79
	P-3	−0.066	−0.246	−0.176	−0.337	−1.27	−0.878	0.74	0.22	0.39
Mercury (μg L^−1^)	P-2	*0*.*982*	*0*.*887*	*0*.*774*	*19.4*	*7.46*	*4.58*	*< 0.001*	*< 0.001*	*<0.001*
	P-3	*0*.*960*	*0*.*876*	*0*.*790*	*17.5*	*9.08*	*6.31*	*< 0.001*	*< 0.001*	*< 0.001*
Organic elements										
PAH (μg L^−1^)	P-2	0.360	0.385	0.430	1.54	1.62	1.78	0.14	0.12	0.10
	P-3	*0*.*736*	*0*.*576*	*0*.*540*	*5.50*	*3.52*	*3.15*	*< 0.001*	*0.002*	*0.004*

^a^The result of the statistics for data from the same research dates (a), for data from piezometers shifted by 1 quarter (b) and two quarters (c)

^b^Italic value of statistics means that the relationship is statistically significant at *p* < 0.05

## Discussion

The average quantity of deposited municipal waste in the examined landfill site over a period of 19 years was considerably lower than the 1600 Mg day^−1^ at a large landfill site in Egypt (El-Salam and Abu-Zuid 2015).

According to Foo and Hameed (2009), a landfill site that has been in operation for over 10 years is classified as an old landfill site, which is reflected in the composition of the leachate.

Analysis of the leachate from the small landfill site conducted over 7 years showed variability of its composition. According to Fatta et al. (1999), the variability of the chemical composition of the leachate depends on many factors, including in particular the original composition of the deposited waste and its various chemical substances, and the chemical and biochemical reactions that may occur when the waste decomposes. Concentrations of Cd and Hg, considered as toxic, (Mor et al. 2006; Fauziah et al. 2013) were lower than those mentioned by Chofqi et al. (2004) and El-Salam and Abu-Zuid (2015). Cadmium is one of the most serious threats to the environment and human health. Vehicular emissions and agricultural activities also increase the concentration of heavy metals, including cadmium (Singh et al. 2016). Moreover, a low average concentration of Cd (1.05 μg L^−1^) contributed to the deterioration of groundwater quality below a landfill site. Higher content of very mobile leachate effluents with Cd concentrations of 30 μg L^−1^ could have contributed to the interaction of landfill waste with groundwater and the migration of pollutants into the aquatic environment (Christensen et al. 1996). The contaminants entering the groundwater under the influence of waste leaching were subject to processes of change. According to Srivastava and Ramanathan (2008), groundwater flow helps in the dispersion and diffusion of leached pollutants in an aquifer system.

Other researcher Tałałaj (2014) showed that a low level of metals followed from the fact that they were released and penetrated the leachate in small quantities only due to processes conducive to immobilisation of metals, such as sorption, chemical precipitation of metals, and higher pH values. The highest level of the reaction in the tested leachate with a pH level at 9.4 only confirmed significant alkalinity, which is not conducive to dissolution of metal ions, contrary to an acidic environment (Schiopu and Gavrilescu 2010). Słomczyńska and Słomczyński (2004) reported that, in landfills exploited for a longer period of time, the alkalinity of the leachate increases up to 8.5 pH. As a comparison, the pH level in other landfill sites was observed at a lower level (Rahim et al. 2010; Brennan et al. 2016). In the examined leachate, the content of zinc was significantly higher: 1.55 mg L^−1^, than its value, ranging between 0.10 and 0.50 mg L^−1^, as specified by Rapti-Caputo and Vaccaro (2006). According to Fauziah et al. (2013), elevated values of contaminant concentrations in the leachate are typical of the first 3–8 years, when the biodegradation occurs fast. On the other hand, according to Kjeldsen et al. (2002), the landfill site under review should be classified as stabilised due to its operation period bring over 10 years.

Deterioration of the quality of the outflowing water in the area of the landfill site was attributable to the elevated value of the electrical conductivity, which was over 25 times higher than that demonstrated by Baun et al. (2004). Karlık and Kaya (2001) recognised groundwater contamination in landfill areas on the basis of an increased EC value.

According to researchers Mor et al. (2006) and Deshmukh and Aher (2016), a high level of electrical conductivity in groundwater is attributable to the impact of a nearby landfill site. Another conductivity index an elevated value was a result of the dependence between the conductivity of the leachate and the conductivity of the outflowing groundwater, which pointed to the fact that, in pace with the increase of conductivity of the leachate by 1 μS cm^−1^, the conductivity of the groundwater below the landfill site grew by 0.217 μS cm^−1^. Other researchers (McNeill 1990; Tezkan 1999) found a relationship between dissolved substances soluble content and EC.

The average value of 10.6 mg L^−1^ total organic carbon in the outflow water was more than twice as high as that reported by Tałałaj and Biedka (2016) and demonstrated significant deterioration of the classification of the quality of the water. A similar value of TOC in the groundwater near the landfill, exceeding 10 mg C L^−1^, was reported by Koda et al. (2017). The increase in the value of the concentration is a consequence of the natural processes occurring in the groundwater and an evident influence of the anthropogenic factor (Huang et al. 2013). In the leachate, the concentration of total organic carbon was elevated as well and amounted to 555 mg L^−1^. According to Anilkumar et al. (2015), the solubility of organic contaminants in waste can be slightly enhanced through the presence of high levels of organic carbon in the leachate. The results of tests confirmed that leachate from the municipal landfill site under review constituted a source of contamination of the water environment, which was also shown by Kanownik and Policht-Latawiec (2016). Other researchers also proved the impact of municipal solid waste landfills on the deterioration of groundwater quality with varying degrees of pollution (Han et al. 2016; Al-Hogaraty et al. 2008).

The presence of such impacts may correspond to an integrated threat on the aquatic environment from pollutants contained in landfill leachate. Researches (De Souza Machado et al. 2016) have illustrated such a threat in the form of a graphic model, which defined the objectives and quality standards of the environment. This model aided in understanding the transport and displacement of contaminants in the environment. It should be noted that potentially toxic cadmium can be transported in dissolved or particulate form, thus playing a significant role in the adsorption, desorption, and dissolution of metals in the sedimentation process (Gonzalez et al. 2007). Generally, transported contaminations in groundwater, with much slower flow than surface water, can enter through to the last waters through an inflow. Usually, groundwater advection to surface water is low; however, the concentration of pollution increases when surface water inflows as a result of percolation through accumulated road sludge, due to sedimentation of pollutants (Förstner and Wittmann 1979). The occurrence such processes indicates elevated values of groundwater pollution.

In addition, analysis of the correlation matrices, which pertained to the data falling below the measurement threshold of the apparatus and which were reduced by one half, demonstrated that the leachate had an influence on the quality of the groundwater below the landfill site with respect to such variables as the contents of Hg and Cr^+6^. Analysis of the correlation matrix showed that the functioning of the small landfill site had an influence on the pollution of the groundwater as a result of the possibly insufficient efficiency of the existing drainage system after 19 years of operation. Rowe (2005) reported a decrease in the efficiency of the leachate system due to clogging caused by the development of microorganisms in the leachate. Other researches, such as Bashir et al. (2009), considered landfill sites as among the main sources of contamination of the surface waters and groundwater. If the leachate is not collected properly, it may infiltrate the soil and reach the aquifer. In turn, Gworek et al. (2016) concluded, on the basis of their research, that the construction of a vertical barrier and drainage system after modernization effectively reduced the spread of metals from the landfill site.

The demonstrated results of the physicochemical analysis of the groundwater and leachate in the area of a small landfill site covered two periods of operation and a period after the site ceased to operate. According to Kjeldsen et al. (2002), closed landfills constitute a serious threat to groundwater for many years due to the fact that the natural decomposition processes of waste in landfill cause groundwater contamination tens of years after the deposition of the waste.

Despite the negative impact of the examined landfill on the quality of the groundwater, a decrease in the correlation between the quality of the groundwater in the piezometers and the leachate should be considered beneficial considering the time shift caused by the migration of contaminants in the land site. This confirms the validity of the recognition of the hydrogeological environment as a natural pollutant migration factor (Alslaibi et al. 2011; Sizirici and Tansel 2015) from the examined landfill site.

The study of leachate from a landfill site in the 1990s showed its interaction with the quality of groundwater in the region. Han et al. (2016) showed that the most intense groundwater pollution occurred in the area of landfills less than 20 years old. The pollutants migrated from the landfill site into the groundwater in the immediate vicinity and within a few hundred metres. The range of the impact was dependent on local geological and hydrogeological conditions, as well as dilution processes, redox reactions, and ion exchange occurring in the soil and water environment (Alslaibi et al. 2011; Banu and Berrin 2015). Outside the landfill site, there was a process of natural self-cleaning of groundwater. Such processes occurred during the movement of pollutants in the groundwater, which changed the quality (Yong et al. 1992; Yong and Mulligan 2004).

## Conclusions

On the basis of hydrochemical analysis of the leachate and groundwater in the area of a small landfill site carried out during the final period of operation of the site, the following conclusions can be drawn:

- The values of total organic carbon in the leachate and in the groundwater below the landfill site were elevated. Also, a significant increase of the total organic carbon in comparison with the value of that indicator in the inflowing waters was observed.
- With regard to a dependence between the leachate and the groundwater, increased conductivity of the leachate was observed with a concurrent increase in the conductivity of the groundwater.
- The increase in the content of cadmium as one of the microelements caused deterioration of the quality of the outflowing waters.
- Ttpdelhe integrated model of threat to the aquatic environment based on the analysis of PEW, TOC, and Cd indicators showed the negative impact of a small-area landfill on groundwater and indirectly on surface waters. The potential extent of this impact depended on local geological and hydrogeological conditions and processes occurring in the soil and water environment.
- The correlation between the physiochemical characteristics of the leachate and water with a time shift attributable to the extended time of migration of the pollution or transport of the mass in a hydrated land site, in spite of the observed drop in the value of Spearman’s correlation factor, should be deemed an important tool allowing for recognition of pollution of the groundwater.
- The interaction between the leachate and groundwater in the area of the landfill site in question proves that the efficiency of the existing drainage system, serving the purpose of discharging waste water to the defensive wells, is insufficient.

## References

[CR1] Ağdağ ON, Sponza DT (2005). Anaerobic/aerobic treatment of municipal landfill leachate in sequential two-stage up-flow anaerobic sludge blanket reactor (UASB)/completely stirred tank reactor (CSTR) systems. Process Biochemistry.

[CR2] Al-Hogaraty EA, Rizk ZS, Garamoon HK (2008). Groundwater pollution of the quaternary aquifer in northern United Arab Emirates. Water, Air, and Soil Pollution.

[CR3] Alslaibi TM, Mogheir YK, Afifi S (2011). Assessment of groundwater quality due to municipal solid waste landfill leachate. Journal of Environmental Science and Technology.

[CR4] Anilkumar A, Sukumaran D, Vincent SGT (2015). Effect of municipal solid waste leachate on ground water quality of Thiruvananthapuram District, Kerala, India. Applied Ecology and Environmental Sciences.

[CR5] APHA (2007). Standard method for the examination of water and wastewater. American public health association.

[CR6] Aziz SQ, Aziz HA, Yuso MS, Bashir MJ, Umar M (2010). Leachate characterization in semi-aerobic and anaerobic sanitary landfills: a comparative study. Journal of Environmental Management.

[CR7] Banu S, Berrin T (2015). Parametric fate and transport profiling for selective ground water monitoring at closed landfills: a case study. Waste Management.

[CR8] Bashir MJK, Isa MH, Kutty SRM, Awang ZB, Aziz HA, Mohajeri S, Farooqi IH (2009). Landfill leachate treatment by electrochemical oxidation. Waste Management.

[CR9] Baun A, Ledin A, Reitzel LA, Bjerg PL, Christensen TH (2004). Xenobiotic organic compounds in leachates from ten Danish MSW landfills—chemical analysis and toxicity tests. Water Research.

[CR10] Brennan RB, Healy MG, Morrison L, Hynes S, Norton D, Clifford E (2016). Management of landfill leachate: the legacy of European Union Directives. Waste Management.

[CR11] Chofqi A, Younsi A, Lhadi EK, Mania J, Mudry J, Veron A (2004). Environmental impact of an urban landfill on a coastal aquifer (El Jadida, Morocco). Journal of African Earth Sciences.

[CR12] Christensen JB, Jensen DL, Christensen TH (1996). Effect of dissolved organic carbon on the mobility of cadmium, nickel and zinc in leachate polluted groundwater. War. Res..

[CR13] Council Directive 1999/31*/*EC of 26 April 1999 on the landfill of waste*,* http://www.odpady.pwr.edu.pl/p/_/84/uchylajaca.pdf, accessed (6.03.2018).

[CR14] De Souza Machado AA, Spencer K, Kloas W, Toffolon M, Zarfl C (2016). Metal fate and effects in estuaries: a review and conceptual model for better understanding of toxicity better understanding of toxicity. Science of the Total Environment.

[CR15] Deshmukh KK, Aher SP (2016). Assessment of the impact of municipal solid waste on groundwater quality near the Sangamner City using GIS approach. Water Resources Management.

[CR16] Dhere AM, Pawar CB, Pardeshi PB, Patil DA (2008). Municipal solid waste disposal in Pune city–an analysis of air and groundwater pollution. Current Science.

[CR17] El-Salam MMA, Abu-Zuid GI (2015). Impact of landfill leachate on the groundwater quality: a case study in Egypt. Journal of Advanced Research.

[CR18] Fatta D, Papadopoulos A, Loizidou M (1999). A study on the landfill leachate and its impact on the groundwater quality of the greater area. Environmental Geochemistry and Health.

[CR19] Fauziah SH, Izzati MN, Agamuthu P (2013). Toxicity on Anabas Testudineus: a case study of sanitary landfill leachate. Procedia Environmental Sciences.

[CR20] Foo KY, Hameed BH (2009). An overview of landfill leachate treatment via activated carbon adsorption process. Journal of Hazardous Materials.

[CR21] Förstner, U., & Wittmann, G.T.W. (1979). Metal pollution in the aquatic environment. *Springer-Verlag*, Berlin Heidelberg New York. ISBN 978–3–642-69385-4.

[CR22] Gavrilescu M (2004). Removal of heavy metals from the environment by biosorption. Engineering in Life Sciences.

[CR23] Gonzalez, J. L., Thouvenin, B., & Dange, C. (2007). Rôle des particules sur le comportement et a speciation de métaux traces: example du cadmium. *La Houille Blanche*, (4), 56–62.

[CR24] Gworek B, Dmuchowski W, Koda E, Marecka M, Baczewska AH, Brągoszewska P, Sieczka A, Osiński P (2016). Impact of the municipal solid waste Łubna Landfill on environmental pollution by heavy metals. Water.

[CR25] Han Z, Ma H, Shi G, He L, Wei L, Shi O (2016). A review of groundwater contamination near municipal solid waste landfill sites in China. Science of the Total Environment.

[CR26] Huang G, Sun J, Zhang Y, Chen Z, Liu F (2013). Impact of anthropogenic and natural processes on the evolution of groundwater chemistry in a rapidly urbanized coastal area, South China. The Science of the Total Environment.

[CR27] Kale SS, Kadam AK, Kumar S, Pawar NJ (2010). Evaluating pollution potential of leachate from landfill site, from the Pune metropolitan city and its impact on shallow basaltic aquifers. Environmental Monitoring and Assessment.

[CR28] Kanownik W, Policht-Latawiec A (2016). Impact of municipal landfill site on water quality in the Włosanka stream. Journal of Ecological Engineering.

[CR29] Karlık G, Kaya AM (2001). Investigation of groundwater contamination using electric and electromagnetic methods at an open waste-disposal site: a case study from Isparta, Turkey. Environmental Geology.

[CR30] Kjeldsen P, Christophersen M (2001). Composition of leachate from old landfills in Denmark. Waste Management & Research.

[CR31] Kjeldsen P, Barlaz MA, Rooker AP, Baun A, Ledin A, Christensen TH (2002). Present and long-term composition of MSW landfill leachate: a review. Critical Reviews in Environmental Science and Technology.

[CR32] Koda E, Miszkowska A, Sieczka A (2017). Levels of organic pollution indicators in groundwater at the old landfill and waste management site. Applied Sciences.

[CR33] Koshy L, Paris E, Ling S, Jones T, BéruBé K (2007). Bioreactivity of leachate from municipal solid waste landfills — assessment of toxicity. The Science of the Total Environment.

[CR34] Laner D, Crest M, Scharff H, Morris JWF, Barlaz MA (2012). A review of approaches for the long-term management of municipal solid waste landfills. Waste Management.

[CR35] Magrinho A, Didelet F, Semiao V (2006). Municipal solid waste disposal in Portugal. Waste Management.

[CR36] Mcneill, J.D. (1990). Use of electromagnetic methods for groundwater studies. In: Ward SH (ed) Geotechnical and environmental geophysics, Vol 1. *Soc Expl Geophys*, Tulsa, pp 191–218.

[CR37] Mor S, Ravindra K, Dahiya RP, Chandra A (2006). Leachate characterization and assessment of groundwater pollution near municipal solid waste landfill site. Environmental Monitoring and Assessment.

[CR38] Rahim BEE, Yusoff I, Samsudin AR, Yaacob WZW, Rafek AGM (2010). Deterioration of groundwater quality in the vicinity of an active open-tipping site in West Malaysia. Hydrogeology Journal.

[CR39] Rapti-Caputo D, Vaccaro C (2006). Geochemical evidences of landfill leachate in groundwater. Engineering Geology.

[CR40] Regadío M, Ruiz AI, de Soto IS, Rastrero MR, Sánchez N, Gismera MJ, Sevilla MT, da Silva P, Procopio JM, Cuevas J (2012). Pollution profiles and physicochemical parameters in old uncontrolled landfills. Waste Management.

[CR41] Regulation of the Minister of Environment of December 21, 2015 on the criteria and method of assessing the status of homogeneous bodies of groundwater (OJ 2016, item 85), http://prawo.sejm.gov.pl/, accessed (6.03.2018).

[CR42] Regulation of the Minister of Environment of November 18, 2014 concerning the Conditions that should be met upon introduction of wastewaters to water or to the ground (OJ 2014, item 1800) http://prawo.sejm.gov.pl/, accessed (6.03.2018).

[CR43] Ritzkowski M, Stegmann R (2007). Controlling greenhouse gas emissions through landfill in situ aeration. International Journal of Greenhouse Gas Control.

[CR44] Rowe RK (2005). Long-term performance of contaminant barrier systems. Geotechnique.

[CR45] Schiopu A, Gavrilescu M (2010). Options for the treatment and management of municipal landfill leachate: common and specific issues. Clean: Soil, Air, Water.

[CR46] Singh, H., Raju, N. J., Gossel, W., & Wycisk, P. (2016). Assessment of pollution potential of leachate from the municipal solid waste disposal site and its impact on groundwater quality, Varanasi environs, India. *Arabian Journal of Geosciences,* 9(2), 131.

[CR47] Sizirici B, Tansel B (2015). Parametric fate and transport profiling for selective groundwater monitoring at closed landfills: a case study. Waste Management.

[CR48] Słomczyńska B, Słomczyński T (2004). Physico-chemical and toxicological characteristics of leachates from MSW landfills. Polish Journal of Environmental Studies.

[CR49] Srivastava SK, Ramanathan AL (2008). Geochemical assessment of groundwater quality in vicinity of Bhalswa landfill, Delhi, India, using graphical and multivariate statistical methods. Environmental Geology.

[CR50] Tałałaj IA (2014). Release of heavy metals on selected municipal landfill during the calendar year. Annual Set The Environmental Protrction.

[CR51] Tałałaj IA, Biedka P (2016). Use of the landfill water pollution index (LWPI) for groundwater quality assessment near the landfill sites. Environmental Science and Pollution Research.

[CR52] Tezkan B (1999). A review of environmental applications of quasi-stationary electromagnetic techniques. Surveys in Geophysics.

[CR53] VanGulck JF, Rowe RK (2004). Influence of landfill leachate suspended solids on clog (biorock) formation. Waste Management.

[CR54] Wiśnios M, Kanownik W, Bogdał A (2015). Hydrochemistry of springs in the Ojcow National Park. Acta Scientiarum Polonorum-Formatio Circumiectus.

[CR55] Xie S, Ma Y, Strong PJ, Clarke WP (2015). Fluctuation of dissolved heavy metal concentrations in the leachate from anaerobic digestion of municipal solid waste in commercial scale landfill bioreactors: the effect of pH and associated mechanisms. Journal of Hazardous Materials.

[CR56] Yong RM, Mulligan CN (2004). Natural attenuation of contaminants in soils. Environment International.

[CR57] Yong RM, Mohamed AMO, Warkentin BP (1992). Principles of contaminant transport in soils. Elsevier..

